# Impact Fracture Simulation of Laminated Glass Based on Thick Shell Elements and a Cohesive Zone Model

**DOI:** 10.3390/ma16216966

**Published:** 2023-10-30

**Authors:** Wei Xia, Zhen Yue, Mengyan Zang

**Affiliations:** 1 School of Mechanical and Automotive Engineering, South China University of Technology, Guangzhou 510000, China; 18289371760@163.com; 2 School of Ocean Engineering and Technology, Sun Yat-sen University, Zhuhai 519000, China; yue255994397@126.com

**Keywords:** thick shell element, cohesive zone model, laminated glass, impact fracture

## Abstract

Laminated glass is extensively used in automotive windshields, making it crucial to have a comprehensive understanding of its fracture mechanism to ensure driver and pedestrian safety in various windshield impact scenarios. Current research on the cohesive zone model of glass impact failure has encountered challenges related to accuracy and computational efficiency. This paper addresses these issues by utilizing the finite element software LS-DYNA, which integrates a cohesive zone model and thick shell (Tshell) elements to simulate and analyze the impact failure process of laminated glass. The combination of Tshell and cohesive elements was validated using a DCB example. Subsequently, the proposed method was applied to simulate the impact damage on an automobile’s front windshield, providing valuable insights from the obtained results. Finally, the influence of curvature, the number of layers, and the thickness ratio of each layer were investigated, leading to some valuable conclusions. Firstly, an increase in the thickness of the upper glass layer correlates with a decrease in the peak acceleration of the dummy-head model due to the ductility of PVB material. Secondly, when a curvature exists, the arched configuration of the windshield promotes higher resistance against impact, consequently leading to increased peak acceleration.

## 1. Introduction

With the rapid growth of the automotive industry, the global number of cars has exceeded 1.3 billion as of the end of 2022. Among the various critical components of automobiles, the windshield plays a pivotal role in ensuring optimal visibility for drivers while assuming significant safety responsibilities [1]. Comprised of two layers of soda-lime glass and a layer of PVB film, the windshield offers remarkable impact resistance and effectively prevents the scattering of glass fragments. Given the widespread use of laminated glass in the automotive sector, it becomes crucial to assess the impact resistance simulation of laminated glass for the safety of both drivers and pedestrians. Due to the inherent brittle failure characteristic of laminated glass, simulating impact failure using continuum-based finite element methods presents a significant challenge. Nevertheless, researchers and engineers worldwide persist in utilizing finite element analysis to simulate the impact failure of laminated glass to meet the demands of engineering applications [2,3]. The development of simulation technology for laminated glass impact failure has progressed through various stages, including the element deleted method (EDM) [4], DEM/FEM [5], cohesive element method [6], and XFEM [7], primarily relying on damage models.

The element deletion method is a widely utilized numerical approach for simulating the failure of laminated glass under impact. This method operates on the principle of establishing a predetermined failure criterion for each element, typically based on its stress or strain characteristics. When the stress or strain of an element surpasses the predefined threshold value due to external loads, the stress or strain of that element is explicitly set to zero, and subsequently, the element is eliminated from the simulation.

Currently, the element deletion method has been widely adopted by various researchers to simulate the impact failure of laminated glass. Different approaches have been explored, such as employing shell elements for both the side glass layers and the intermediate PVB layer [8], utilizing membrane elements for the intermediate PVB layer while employing hexahedral elements for the side glass layers and intermediate PVB layer, and establishing shared-node or tied connections between the glass layer elements on both sides and the intermediate PVB layer elements [9]. In [4], it was proposed that the elements should reach a threshold capacity in the local region before failure occurs. Subsequently, crack initiation and propagation are governed by the maximum stress criterion. The effectiveness of this criterion was validated through tests conducted on plane and curved laminated glass specimens. However, Ref. [10] demonstrated that this numerical approach may not perform optimally when the model contains highly detailed elements.

In order to effectively describe the dispersion of crack growth, Ref. [11] initially proposed using the discrete element method to study the impact failure characteristics of laminated glass. In addition, scholars around the world have also used this method to simulate the failure and interfacial debonding of quartz glass [12] or composite materials [13]. Because the discrete element method is based on the premise of small deformations, it cannot effectively describe the mechanical properties of large deformations of PVB films in the middle layer. Therefore, Ref. [14] proposed a method of coupling discrete elements with finite elements (finite element simulation is used for the intermediate PVB film, and discrete element simulation is used for the glass on both sides, which are tied to the PVB film) to simulate and analyze the dynamic corresponding process of laminated glass under the impact of a steel ball.

However, the discrete element method suffers from the issue of low computational efficiency. In order to address this limitation, Ref. [15] introduced a novel approach that couples discrete elements with finite elements. This technique aims to enhance the computational efficiency of the discrete element method. In the proposed numerical method, the initial representation of the laminated glass plate utilizes the finite element method. However, during the process of impact failure, the finite element representing the glass, which experiences significant deformation, is automatically replaced with a discrete element representation.

The extended finite element method (XFEM) is a novel numerical algorithm introduced by Belytschko and Black, building upon Melenk and Babuska’s element decomposition method, which aims to effectively simulate crack initiation and propagation [7]. By utilizing the element decomposition technique, the XFEM not only addresses the issue of discontinuous displacement fields encountered in traditional finite element methods but also eliminates the need to predefine the crack initiation position. This characteristic makes it more convenient to conduct mesh refinement and update investigations within the XFEM framework.

Consequently, in conjunction with drop-weight impact testing, Ref. [16] employed the XFEM to investigate the failure characteristics of laminated glass subjected to low-speed impacts and analyzed the factors influencing crack growth in the glass. However, it should be noted that the XFEM may not accurately depict crack intersections in the context of impact failure response in laminated glass panels.

The application of cohesive elements in the study of laminated glass impact failure has shown significant advantages in various aspects. Compared to the discrete element method, the cohesive element method offers lower computational requirements while providing more accurate computational results. The cohesive model was initially proposed by [17,18], and it has emerged as one of the most effective approaches for simulating material failure. It has found wide application in areas such as the interlayer debonding [19] and fracture analysis [18] of composite layers.

The fundamental concept of the cohesive model is to assume a gradual opening process at the crack tip. It involves controlling a stress–separation curve to represent the material behavior by inserting zero-thickness elements between the common surfaces of finite elements. This process allows for the transition of a material from a continuum to a discontinuum state. Depending on the specific insertion methods employed, the cohesive model can be broadly classified as intrinsic or extrinsic.

Indeed, computational efficiency is a significant challenge when utilizing cohesive elements. Many research efforts have been dedicated to improving computational efficiency from various perspectives. Simply reducing the mesh density can adversely affect the accuracy of crack representation and distort the results [20]. To address this issue, Ref. [21] proposed the use of higher-order cohesive elements or an increased number of integration points within cohesive elements to more effectively capture the discontinuous displacement field associated with cracks. On the other hand, some researchers have opted for the use of extrinsic cohesive elements to reduce computational requirements. This can be achieved through selective activation or dynamic insertion techniques [22]. In addition, peridynamics [23], which, due to its mesh-free peculiarity, can more easily simulate crack propagation under large deformation, XFEM coupled with peridynamics [24], which use FEM to reduce computation, employ the PD theory only in a small area where steep strain gradients or discontinuous displacement fields are expected, and the phase-field approach [25], proposed in recent years, also have their own advantages in the authenticity of the simulation.

As research progresses, scholars have increasingly shifted their focus from intrinsic/extrinsic cohesive elements to the computational elements themselves. One approach involves the use of incompatible elements, which can enforce a shear stress of zero in the thickness direction, thereby eliminating shear locking. For instance, ref. [26] employed shell elements to simulate the impact on laminated glass, whereas [27] utilized solid-shell elements for similar investigations. However, a limitation of these methods is that they cannot be implemented in commercial software.

At present, the Tshell element has been introduced by LS-DYNA; however, its application in simulating the impact damage of laminated glass is yet to be explored. The derivation of the Tshell element’s equation is based on the co-rotational coordinate system, whereas the strain matrix is obtained by utilizing the Taylor expansion at the element’s center point. Given its capability to overcome shear-locking problems and provide faster computational speeds, the Tshell element has garnered considerable interest.

This paper presents an innovative application of the Tshell element to simulate the impact failure of laminated glass. Initially, the validity of combining Tshell and cohesive elements is verified using a DCB (Double Cantilever Beam) test example. Subsequently, a drop-weight impact test is performed to simulate the impact failure of a laminated glass plate, evaluating the effectiveness of the proposed method in this specific area.

Furthermore, the investigation in this study is extended by applying the proposed method to simulate the impact damage of an automobile’s front windshield. The experiments conducted in this study yield promising results and exhibit considerable potential for engineering applications. Additionally, several parameter studies are conducted to explore the capabilities and limitations of the proposed method in further detail.

This research not only demonstrates the practicality and efficacy of utilizing Tshell elements in the simulation of impact failure in laminated glass structures but also yields valuable insights for the field. The findings have implications for the future design and analysis of laminated glass components subjected to impact loading.

The impact resistance of automobile windshield glass is governed by various factors, including the energy release rate, properties of the polyvinyl butyral (PVB) material, and thickness ratio of each layer of the windshield glass. Furthermore, the number of layers and the curvature of the windshield also play crucial roles in determining the impact resistance and energy absorption performance. However, previous studies have provided limited information on these aspects.

In order to address the research gap, the present study undertakes parameterized investigations on the thickness ratio of each layer of windshield glass, the number of layers, and the curvature. Through systematic variations of these parameters, the objective is to examine the individual and combined impacts on the impact resistance and energy absorption capacities of the windshield glass.

Through this comprehensive analysis, valuable insights into the optimization of these design parameters for enhancing the impact resistance and safety of automobile windshield glass are provided. The findings from these parameterized studies will be an invaluable resource for engineers and researchers in the field, aiding in the development of improved windshield glass designs.

This paper is organized as follows. In Section 2.1 and Section 2.2, the principles of the Tshell element and intrinsic cohesive model are introduced, respectively. In Section 3, a DCB example is constructed to verify the effectiveness of the combination of the Tshell and cohesive elements. In Section 4, the drop-weight impact of laminated glass plate is simulated. In Section 5, a Tshell–cohesive element is applied to the impact damage process of automobile windshield glass. In Section 6, parametric analysis is carried out, and the final summary is provided in Section 7.

## 2. Methodology

### 2.1. Tshell Element

In this article, the Tshell element is primarily utilized for several reasons. Firstly, when compared to shell elements, the three-dimensional element effectively captures crack growth in the thickness direction, and its calculation accuracy is higher than that of shell elements. Additionally, the Tshell element offers significant advantages over solid elements. Through a special treatment of the strain matrix, shear locking is eliminated, enabling the Tshell element to achieve comparable accuracy while utilizing fewer elements. Moreover, the fully integrated Tshell element utilizes a co-rotational coordinate system [28] designed for small deformations and large rotations, which enables efficient stress updating. This approach has been shown to be more efficient than the traditional updated Lagrange method, resulting in a greatly improved calculation speed.

#### 2.1.1. Shear-Locking Problem

The internal forces of elements are obtained by integrating the strain matrix and Cauchy stress tensor before numerical integration, namely:

(1) $$\;f^{in}=\int TBdV$$

In the traditional solid element, B is obtained through the partial derivative of the form function and the Jacobian matrix transformation, namely:

(2) $$\;B_{i}=J^{-1}LN_{i}$$

Unlike the strain operator of the standard solid element, the strain operator of the Tshell element is expanded into a Taylor series about the center of the element, and the shear strain component of the strain matrix can be written in the co-rotational coordinate system as:

(3) $$\;B_{xy}^{dev}\left(\xi ,\eta ,\zeta \right)=B_{xy}^{dev}\left(0\right)+B_{xy,\xi }^{dev}\left(0\right)\xi$$

(4) $$\;B_{yz}^{dev}\left(\xi ,\eta ,\zeta \right)=B_{yz}^{dev}\left(0\right)+B_{yz,\zeta }^{dev}\left(0\right)\zeta$$

(5) $$\;B_{xy}^{dev}\left(\xi ,\eta ,\zeta \right)=B_{xy}^{dev}\left(0\right)+B_{xy,\eta }^{dev}\left(0\right)\eta$$

where there is only one linear term left in the shear-strain component, that is, the shear stiffness is constant, which eliminates the modes leading to shear locking and improves the convergence rate in implicit analysis.

**Figure 1:**
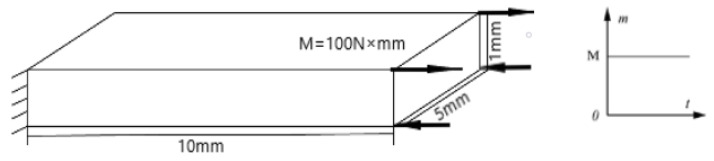
Schematic diagram of the cantilever plate model.

To provide a more direct illustration of the problem, a set of simple cantilever plate-bending examples are calculated to compare the ability of two elements to resist shear locking. The model, as shown in Figure 1, consists of a cantilever plate with one end fully restrained and the other end subjected to a continuous torque of 100 N×m. The material properties used in the calculations are as follows: E is 210 GPa, the density is 7.9 kg/m${}^{3}$, and *v* is 0.3.

In order to model the bending moment, it is considered as a uniform load applied symmetrically on both sides of the free end, with a load of 20 N/mm. Initially, a very fine model consisting of 80 × 40 × 8 cubic fully integrated solid elements is established to obtain the reference results. Subsequently, relatively coarse models of 4 × 4 × 2, 16 × 16 × 8 solid elements, and 4 × 4 × 2 Tshell elements are created.

**Figure 2:**
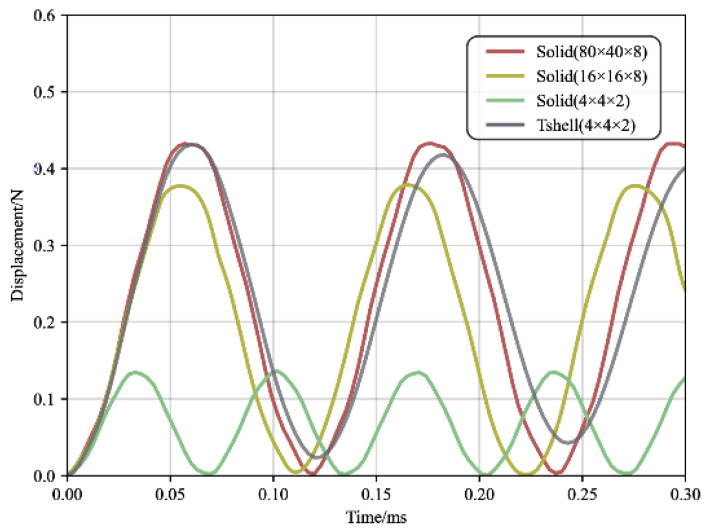
Displacement–time curve of cantilever plate.

The three models mentioned above, with fewer elements, were deliberately designed to have the same width-to-thickness ratio of 5. The displacement of the free end’s center in the cantilever plate is shown in Figure 2. It can be observed that the maximum deflection obtained from the numerical model with 16 × 16 × 8 solid elements is lower than the reference value obtained from the very fine model. Furthermore, the Tshell element yields calculation results consistent with the reference value obtained using 4 × 4 × 2 elements.

#### 2.1.2. Co-Rotational Coordinate System

The co-rotational coordinate method was initially proposed in the 1970s by Wempner, Belytschko, and Hsieh as a means to address practical problems involving large rotations, displacements, and small strains. Over the years, this method has undergone significant developments and has proven to be more efficient in handling such issues. The fundamental concept behind this method involves dividing the deformation of an element into three stages—translational, rotational, and deformation. Within the co-rotational coordinate system, the Cauchy stress tensor is transformed into the co-rotational Cauchy stress tensor. As this is an objective quantity, there is no need to rotate stress using the Jouman stress rate during stress updating, thereby accelerating the calculation speed [29].

To present the principles in greater detail, a hypothetical scenario is considered, where the stresses and strains from the preceding step are already known. Since the measurement of stress and strain in the co-rotational coordinate system is objective, the focus is on the calculation of the incremental strain within the time period [$t_{n},t_{n+1}$]. Consequently, the stress can be updated using a radial regression algorithm. In this context, $\Omega_{n}$ denotes the configuration at time $t=t_{n}$, and $\Omega_{n+1}$ denotes the configuration at time $t=t_{n+1}$. All kinematics are computed based on the preceding configuration.

(6) $$\;{\hat{x}}_{i}=R_{i}x_{i}$$

In the Tshell element, the strain increment is represented by the midpoint ($t_{n+1/2}$), i.e,

(7) $$\;\Delta \varepsilon =\int_{t_{n}}^{t_{n+1}}\hat{d}d\tau =N$$

(8) $$N=\frac{1}{2}\left[\partial {\hat{u}}^{def}/\partial {\hat{x}}_{n+1/2}+{\left(\partial \Delta {\hat{u}}^{def}/\partial {\hat{x}}_{n+1/2}\right)}^{t}\right]$$

In order to solve the aforementioned equation, it is necessary to obtain the configuration of the element at time $t_{n+1/2}$. By making the assumption that the velocity remains constant throughout the time period $\left[t_{n},t_{n+1}\right]$, the following can be derived:

(9) $$\;{\hat{x}}_{n+1/2}=R_{n+1/2}x_{n+1/2}=\frac{1}{2}R_{n+1/2}\left(x_{n}+x_{n+1}\right)$$

**Figure 3:**
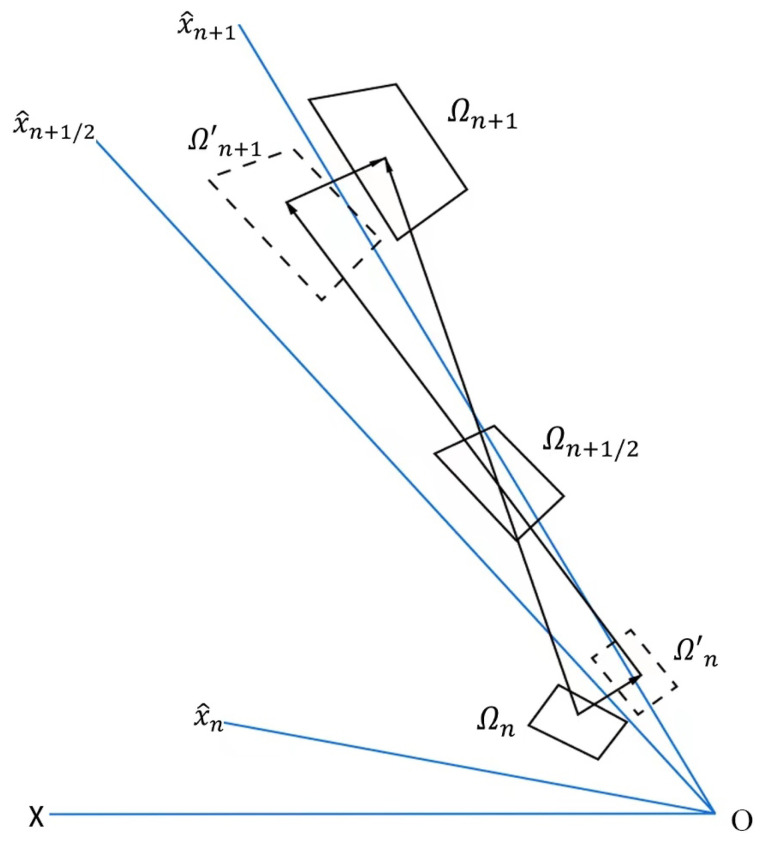
Co-rotational coordinate system.

Similar to polar decomposition, the deformation increment can be decomposed into the addition of pure deformation and pure rotation. Let Δu be the displacement increment in the period $\left[t_{n},t_{n+1}\right]$, and then $\Delta u=\Delta u^{def}+\Delta u^{rot}$. The deformation part also includes translational displacement without strain. To obtain the displacement at $t_{n+1/2}$, the inverse operation is performed from $\Omega_{n}$ to $\Omega_{n+1}$ for rigid body rotation displacement. Two virtual configurations, $\Omega_{n}^{\prime }$ and $\Omega_{n+1}^{\prime }$, are defined, with their positions shown in Figure 3.

In the process from $\Omega_{n}$ to $\Omega_{n+1}$ and from $\Omega_{n}^{\prime }$ to $\Omega_{n+1}^{\prime }$, the object goes through two equivalent rigid rotations about the point O. The x-coordinate remains the same, and the magnitude of the rotational displacement can be calculated using the following equation:

(10) $$\;\Delta u^{rot}=x^{\prime }-x=R_{n+1/2}^{t}\hat{x}-x$$

Therefore, its total rotational displacement increment can be expressed as:

(11) $$\;\Delta u^{rot}=\Delta u_{1}^{rot}+\Delta u_{2}^{rot}=\Delta u-R_{n+1/2}^{t}\left({\hat{x}}_{n+1}-{\hat{x}}_{n}\right)$$

The displacement increment in [$t_{n},t_{n+1/2}$] can be obtained as follows:

(12) $$\;\Delta {\hat{u}}^{def}=\Delta u-\Delta u^{rot}=R_{n+1/2}^{t}\left({\hat{x}}_{n+1}-{\hat{x}}_{n}\right)$$

Therefore, the deformation displacement increment under the co-rotational system at $t_{n+1/2}$ is

(13) $$\;\Delta {\hat{u}}^{def}=R_{n+1/2}\Delta u^{def}={\hat{x}}_{n+1}-{\hat{x}}_{n}$$

The change in displacement is the difference between the coordinates at time n+1 and the coordinates at time n in the co-rotational system. Note that the transformation matrix cancels out.

Where there is a displacement increment, a strain increment can be obtained, and a stress increment is also bound to the midpoint, which can be calculated by the radial regression algorithm. The overall strain tensor and stress tensor can be calculated using the following formula:

(14) $$\;{\hat{\varepsilon }}_{n+1}={\hat{\varepsilon }}_{n}+\Delta \hat{\varepsilon }$$

(15) $$\;{\hat{\sigma }}_{n+1}={\hat{\sigma }}_{n}+\Delta \hat{\sigma }$$

The calculated stress and strain tensors are noteworthy, as they are linked to the current configuration and defined within the current co-rotational coordinate system. By applying tensor transformation rules, one can acquire the strain and stress tensors corresponding to the global coordinate system.

### 2.2. Intrinsic Cohesive Element

**Figure 4:**
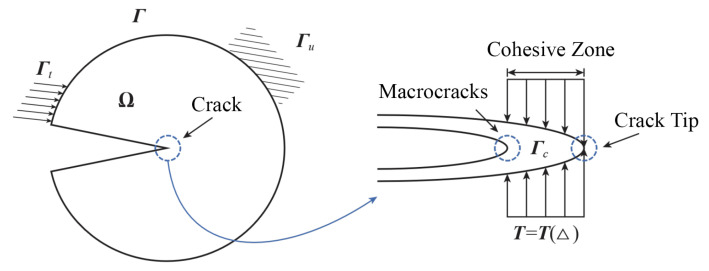
Cohesive model.

As depicted in Figure 4, the general problem domain Ω consists of cracks and boundaries Γ, where various boundary conditions, such as velocity, displacement, stress, and constraints, are simultaneously applied. These boundary conditions are represented by the subsets $\Gamma_{t}$ and $\Gamma_{n}$.

The cohesive model is a simplified representation that assumes a cohesive zone at the crack tip, even though, in reality, there is no crack present. Instead, the structure is in a state transitioning from unbroken to near breakage, or the material has already undergone yielding. The stress distribution within the cohesive region can be described by the following equation:

(16) T=T(Δ)

where *T* stands for the cohesive force, and Δ represents the relative displacement of points at the crack interface in this region. Since the relative displacement Δ of the point at the crack tip is zero, the cohesive force *T* of the point is zero, thus avoiding the problem that the stress at the crack tip tends to infinity.

**Figure 5:**
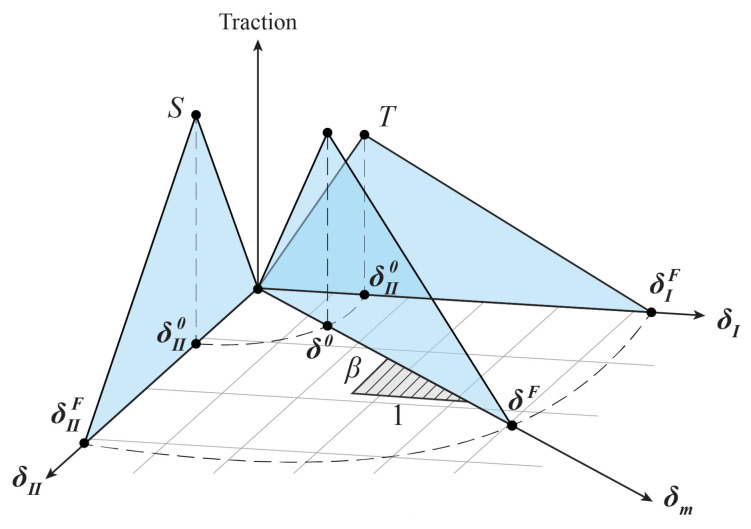
Mixed-mode tractor-separation criterion.

The cohesive element has various constitutive models. In this paper, the cohesive element adopts the bilinear tractor-separation criterion (TSL), as shown in Figure 5. The TSL satisfies the following equation:

(17) $$\;E^{\prime }=Q_{MAX}/L$$

There is another important parameter in the model: the energy release rate. It represents the amount of energy needed for a structure to break down. In theory, the value is equal to the area of the blue line in the graph:

(18) $$\;G_{C}=1/2uQ_{MAX}$$

In practical engineering applications, due to the very complex loading mode, the cracks generated in this way often contain the fracture forms of mixed mode that should be introduced. As shown in Figure 5, in the cohesive model, the total relative displacement under the mixed mode satisfies the following equation:

(19) $$\;\delta_{m}=\sqrt{\delta_{I}^{2}+\delta_{II}^{2}}$$

Among them, the equation $\delta_{I}=\delta_{II}$ is satisfied in the type I fracture form. In the type II fracture form, the equation $\delta_{m}=\sqrt{\delta_{I}^{2}+\delta_{II}^{2}}$ is satisfied. The initial displacement of mixed-mode damage can be expressed by the following equation:

(20) $$\;\delta^{0}=\delta_{I}^{0}\delta_{II}^{0}\sqrt{\left(1+\beta^{2}\right)/\left({\left(\delta_{II}^{0}\right)}^{2}+{\left(\beta \delta_{I}^{0}\right)}^{2}\right)}$$

where $\delta_{I}^{0}=T⁄EN$ and $\delta_{II}^{0}=S/ET$, respectively, represent the initial displacement of a single fault type, $\beta =\delta_{II}/\delta_{I}$. In addition, the BK criterion (Benzeggagh–Kenane law) is selected in this paper to define the failure process of cohesive elements in structures, so the ultimate displacement $\delta^{F}$ of the final mixing mode satisfies the following equation:

(21) $$\;\delta^{F}=\frac{2(G_{CI}+\left(G_{CII}-G_{CI}\right))}{\delta^{0}{\left(1/\left(1+\beta^{2}\right)EN^{\gamma }+\beta^{2}/\left(1+\beta^{2}\right)ET^{\gamma }\right)}^{\left(1⁄\gamma \right)}}K$$

(22) $$\;K={\left(\frac{\beta^{2}ET}{EN+\beta^{2}ET}\right)}^{\left|XMU\right|}$$

where γ represents the BK criterion of the additional index, and the recommended value GAMMA = 1.0 (default) or γ.

## 3. The Double Cantilever Beam Test

Due to no study having been conducted on the combination of Tshell and cohesive elements, it is necessary to construct a DCB example to independently verify the effectiveness of the combination of Tshell and cohesive elements.

### 3.1. Example Description

**Figure 6:**
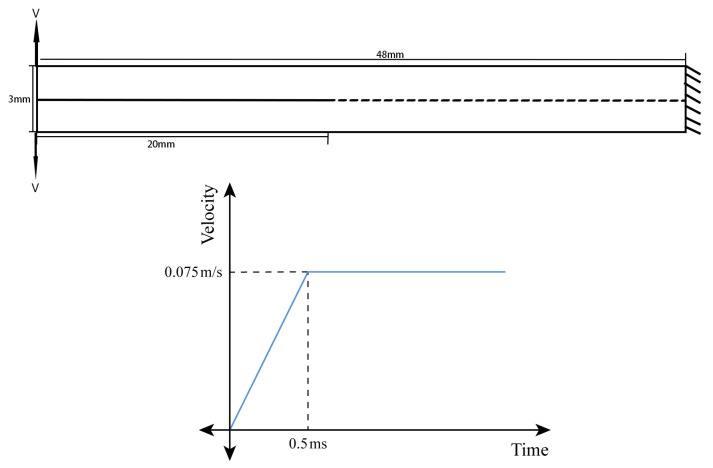
Schematic diagram of DCB example.

**Figure 7:**
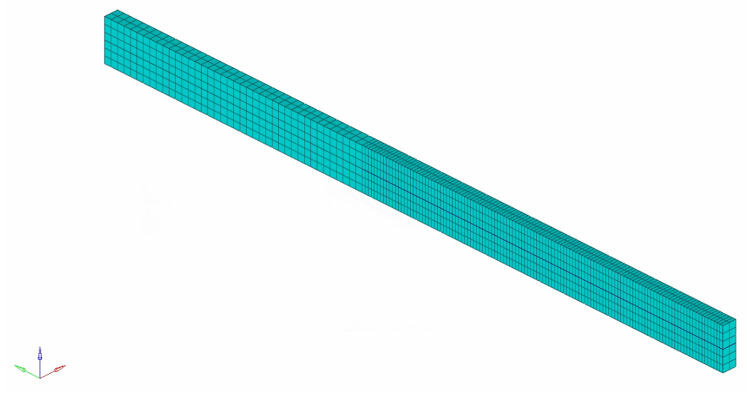
Finite element meshes of DCB example.

The specific details of the double cantilever beam (DCB) example are illustrated in Figure 6, and the finite element meshes are shown in Figure 7. The right end of the beam is fully constrained, whereas the left end applies the load through displacement control. The beam has a cross-sectional area of 48 × 3 mm${}^{2}$, and the length of the prefabricated crack is 20 mm. The Tshell element is employed to discretize the beam, and a cohesive element with zero thickness is inserted along the potential crack path. Regarding the material parameters, v=0.25, E=135 GPa, $C_{t}^{max}=C_{n}^{max}=57$ MPa, $G_{I}=G_{sc}=0.281$ N/mm, and $k=110^{5}$ MPa/mm.

### 3.2. Numerical Results

**Figure 8:**
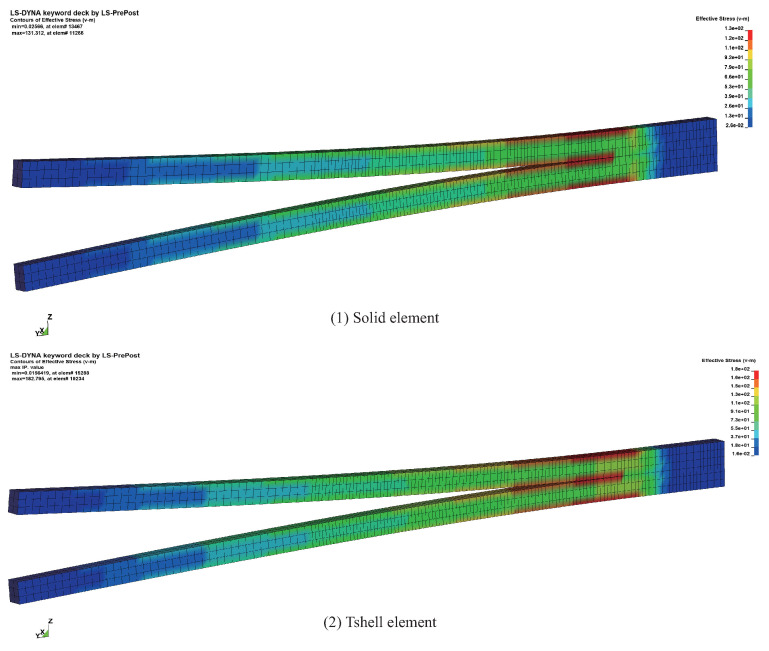
The stress results of DCB examples.

**Figure 9:**
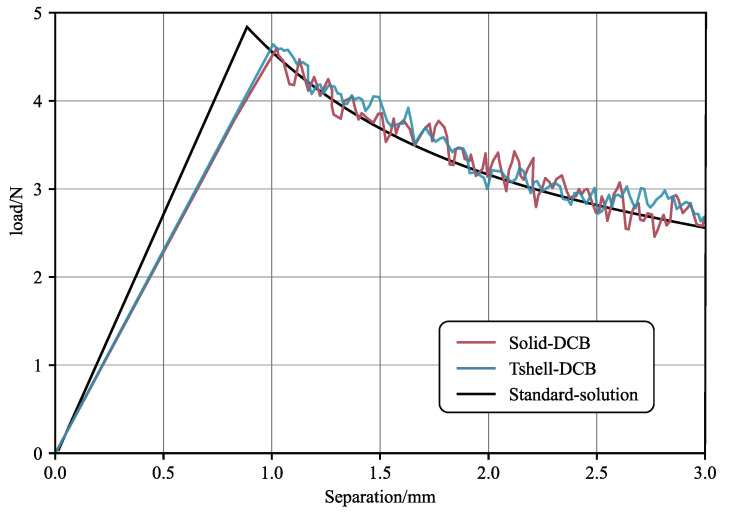
Comparison of separation and load results in DCB examples.

As shown in Figure 8 and Figure 9, it can be observed that the computed results of the example utilizing the Tshell element are in excellent agreement with the computed results obtained from the solid element and the analytic solution. This confirms that the combination of the Tshell and cohesive elements exhibits stable and reliable performance, thereby providing a necessary foundation for the subsequent simulations.

## 4. Simulation of Laminated Glass Plate Drop-Weight Impact

### 4.1. Laminated Glass Plate Drop-Weight Impact Experimental Apparatus

To affirm the viability of the proposed methodology for investigating failure caused by impact in laminated glass, a sophisticated drop-weight impact experimental apparatus has been meticulously devised and implemented. This apparatus has been exclusively tailored for conducting experiments on laminated glass plates [6].

The apparatus consists of the following components: a cylindrical steel impactor with an embedded acceleration sensor, an electromagnet device, a rubber support pad measuring 80 mm × 10 mm × 4 mm in length, and the laminated glass plate test specimen with dimensions of 200 mm × 80 mm × 5.76 mm. The test specimen comprises a middle PVB film with a thickness of 0.76 mm, and upper and lower glass plates with thicknesses of 2 mm and 3 mm, respectively.

**Figure 10:**
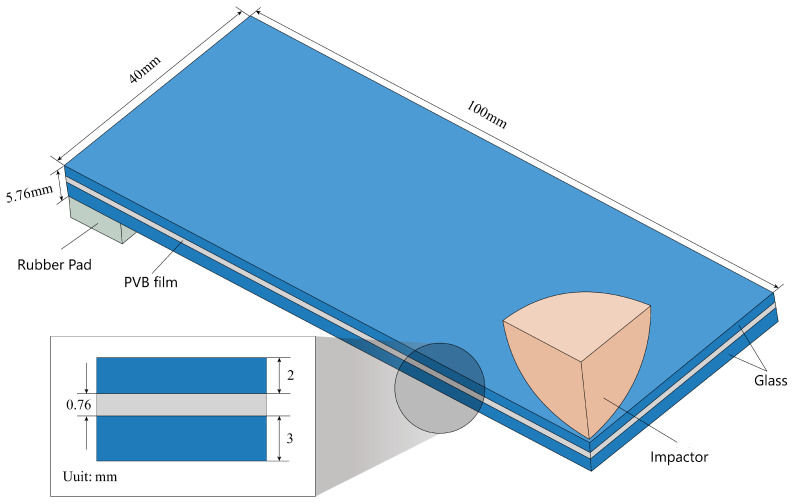
Schematic diagram of drop-weight impact finite element model.

The drop-weight impact test consists of the following steps: Firstly, the laminated glass plate test specimen is placed on the rubber pad positioned 2.3 m below the electromagnet device on the support frame. A mass of 144 grams is adhered to the electromagnet device in its current state. Once the electromagnet is powered off, the drop-weight impactor loses its magnetism and freely falls. The impactor strikes the center of the laminated glass plate test specimen, causing it to fracture, as shown in Figure 10. Additionally, the built-in acceleration sensor in the drop-weight impactor records the acceleration history curve of the impactor during the impact and failure process of the laminated glass plate.

### 4.2. PVB Material Model

The intermediate layer’s PVB film is primarily utilized in the production of laminated glass. It is a polymer material that offers impact resistance, good light transmittance, high mechanical strength, and heat and cold resistance. Moreover, it serves as the optimal bonding material for laminated glass manufacturing. In the context of impact damage to automotive glass, the PVB film plays a crucial role. It not only absorbs energy but also adheres to a significant amount of glass fragments to prevent peeling and reduce the risk of secondary injuries caused by glass splatters to pedestrians and drivers. Hence, the mechanical properties of the PVB film have a substantial influence on the impact failure behavior of laminated glass plates. As one of the pivotal components of laminated glass plates, in-depth research and analysis of its mechanical properties are essential.

In 1940, Mooney conducted a series of tests to describe the large deformation of rubber materials and proposed the strain energy function [22]. It is important to note that the constitutive model for rubber hyperelastic materials significantly differs from that of linear elastic materials. In LS-DYNA, the Mooney–Rivlin model, a hyperelastic material model, is employed to describe the PVB interlayer. The strain energy density function, W, can be defined as follows:

(23) $$\;W\left(I_{1},I_{2},I_{3}\right)=A\left(I_{1}-3\right)+B\left(I_{2}-3\right)+M$$

(24) $$\;M=C\left(I_{3}^{-2}-1\right)+D{\left(I_{3}-1\right)}^{2}$$

where

(25) C=0.5A+B

(26) $$\;D=\frac{A(5v-2)+B(11v-5)}{2(1-2v)}$$

(27) $$\;I_{1}=tr\left(R\right);I_{2}=1/2\left(I_{1}^{2}-tr\left(R^{2}\right)\right);I_{3}=det\left(R\right)$$

### 4.3. Material Definition and Model Description

The material models utilized for each component in the model, along with their corresponding material parameters, are provided in Table 1. The Tshell element is set to ELFORM = 2, with eight integration points, consistent with the fully integrated hexahedron element. The ELFORM of the *SECTION SOLID keyword is set to 19 to define the cohesive element with a 4-point integration. The tensile strength of the glass is defined as 60 MPa, and G in the normal and tangential directions is set to 10/50 N/m. In the case of applying a symmetric boundary condition to the symmetric surface of the finite element model, the initial velocity of the impactor upon contact with the laminated glass is set to 6.7 m/s.

### 4.4. Analysis of Simulation Results

**Figure 11:**
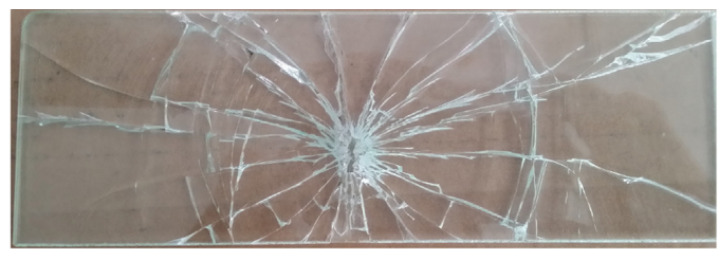
Experimental phenomena of impact failure.

**Figure 12:**
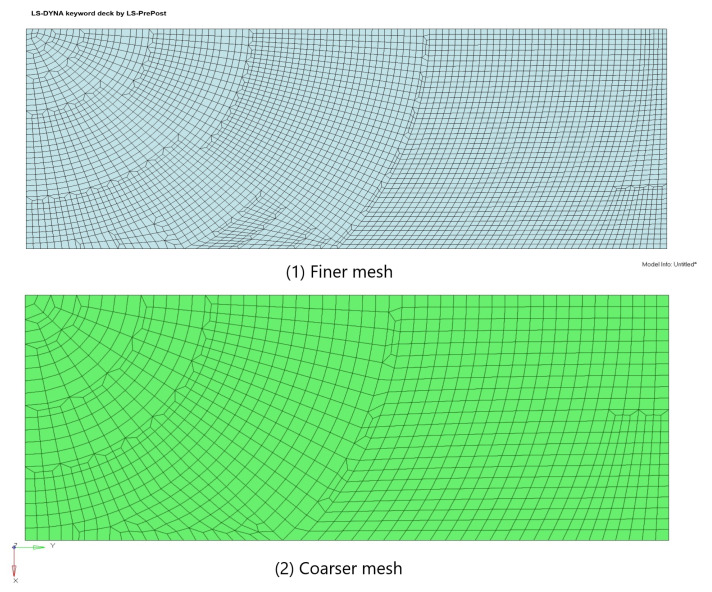
Grid diagram of drop-weight impact finite element model.

**Figure 13:**
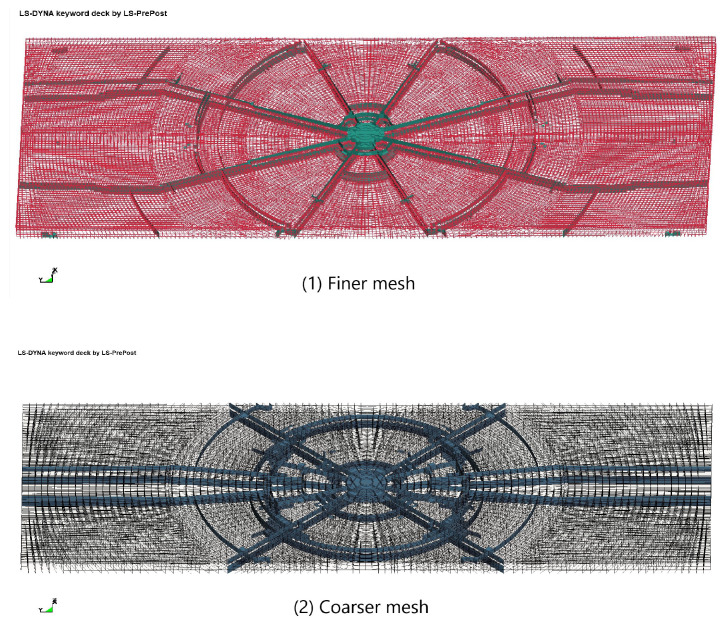
Impact failure phenomenon of laminated glass plate.

When analyzing the experimental findings from the drop-weight impact test, as illustrated in Figure 11, two types of cracks, namely circumferential and circumferential, were observed. In order to accurately capture the circumferential crack, a simulation mesh was established, as depicted in Figure 12. The computational results depicting the crack propagation are presented in Figure 13.

In previous modeling studies that utilized solid elements, denser meshes were required to maintain accuracy. However, these denser meshes also necessitated the use of more layers. In this paper, to address this issue and eliminate shear locking, the Tshell element was employed. As a result, the model mesh was doubled, and the number of mesh layers was halved, as illustrated in Figure 12. The corresponding calculation results are presented in Figure 13.

In order to enhance the scientific nature of this paper, a mesh quality analysis was carried out. For the Jacobian ratio, conditions greater than 0.7 were considered to be of good quality. We utilized the coarse- and fine-mesh models from the drop-weight impact test example in this analysis. For the fine-mesh model, the aspect ratio was about 4.9, and the Jacobian rate was around 0.97. For the coarse-mesh model, the aspect ratio was about 3.01, and the Jacobian rate was around 0.96. Based on previous research, aspect ratios of about 4.9 and Jacobian rates of around 0.97 were considered acceptable. So, the mesh size set in this study was reasonable.

**Figure 14:**
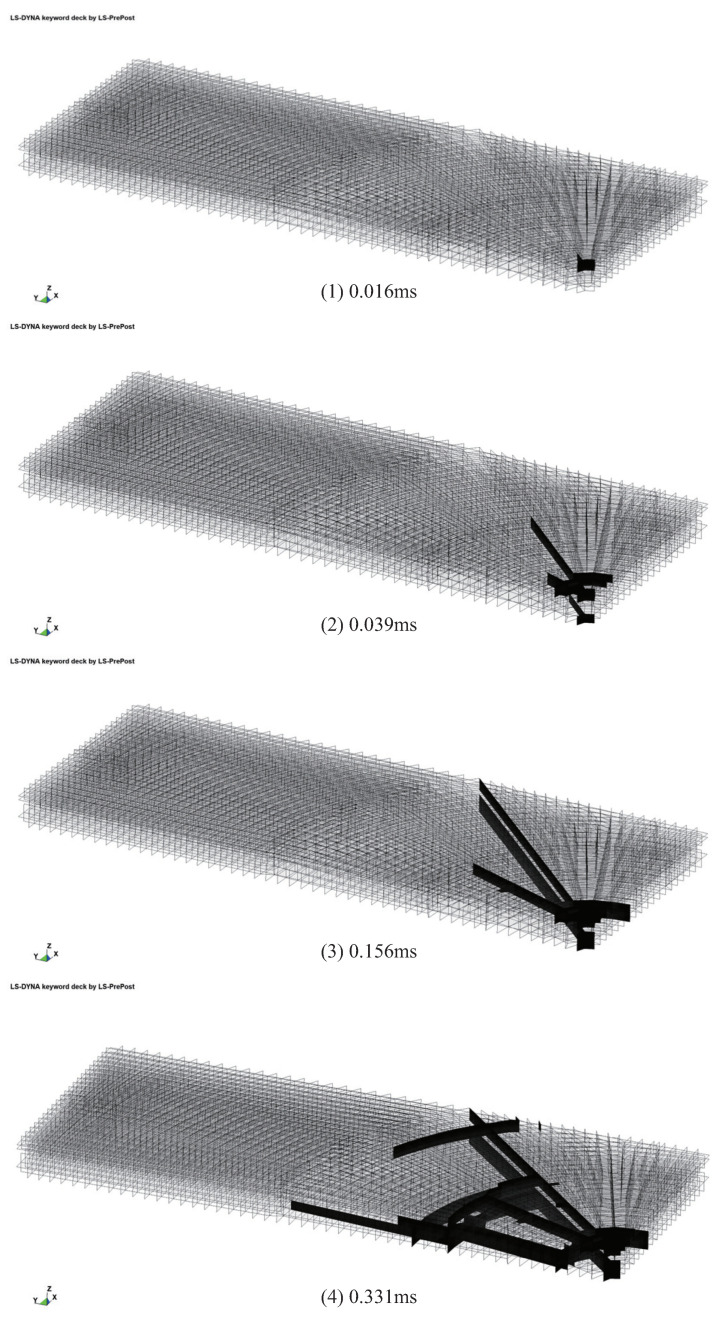
Partition diagram of laminated glass cracking.

Figure 14 illustrates the progressive cracking behavior of the laminated glass plate at different time intervals. The initiation of cracks in the upper-layer glass plate can be observed at 0.016 ms, followed by the emergence of cracks in the lower-layer glass plate at 0.039 ms. Between 0.039 ms and 0.156 ms, both upper- and lower-layer cracks propagated in a parallel plane direction, resulting in the formation of radial cracks. Subsequently, at 0.331 ms, circumferential cracks perpendicular to the radial cracks began to manifest. This cracking pattern is consistent with the computational results obtained using solid elements, thereby substantiating the efficacy of Tshell elements in accurately calculating the impact-induced damage in glass materials.

To validate the effectiveness of the Tshell element in simulating the drop-weight impact on glass plates, a comparison was performed between the experimental acceleration–time history curve of the impactor and the simulation results. Remarkably, the drop-weight acceleration curve obtained using the Tshell element exhibited good agreement with the experimental curve. This serves as evidence of the efficacy of the Tshell–cohesive element in accurately simulating the impact of the drop weight on glass plates.

**Figure 15:**
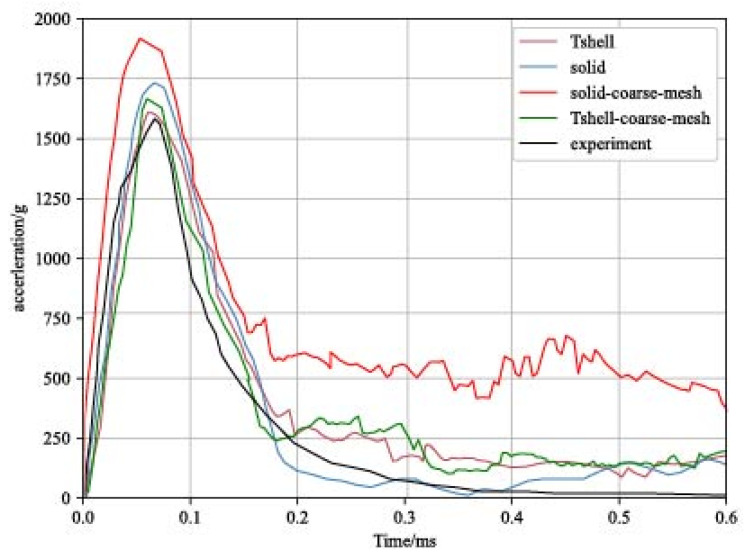
Experimental and simulation acceleration–time history curve of the impactor.

The advantages of the Tshell element in terms of acceleration calculation are clearly demonstrated in Figure 15, which highlights the significant benefits of the Tshell element, indicating that the model achieved the desired accuracy with a reduced mesh, effectively overcoming shear-locking issues. Moreover, it is evident that the Tshell element exhibited lower mesh sensitivity compared to the solid element. This means that the peak acceleration was less influenced by the mesh refinement when using the Tshell element. In other words, the accuracy of the results was less dependent on the mesh resolution.

**Figure 16:**
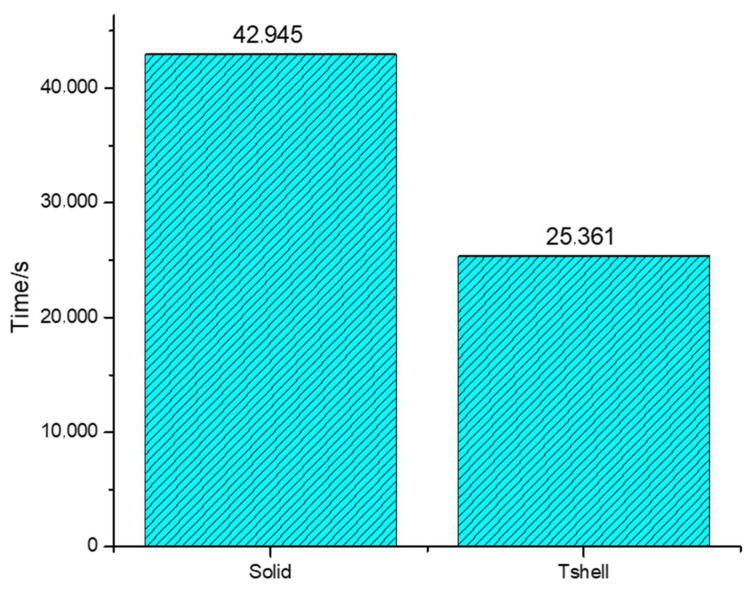
Comparison of the calculation times of the drop-weight impact model.

Furthermore, a comparison between the computation times of the Tshell element and the solid element is presented in Figure 16. The results reveal that the Tshell element outperformed the solid element in terms of computational efficiency, making it a more practical and valuable choice for engineering applications.

## 5. Application in the Windshield Impact Test

### 5.1. Introduction of Experimental Equipment

The Automotive Collision Laboratory of Tsinghua University has designed and developed a test system device for automobile windshield glass, specifically focusing on impact tests using an adult dummy-head module [24]. This test system consists of eight main components, including a buffer system, high-speed camera, dual photoelectric test system, adult dummy-head module, pedestrian collision test launch pad, automobile windshield specimen, windshield bench system, and acceleration sensor. During these impact tests, the adult head-shaped impactor, under the influence of the pedestrian impact test pad, is subjected to different impact angles (60–90${}^{\circ }$) and different impact velocities (ranging from 6.6 m/s to 11.2 m/s), targeting the geometric center of the front windshield of the vehicle.

**Figure 17:**
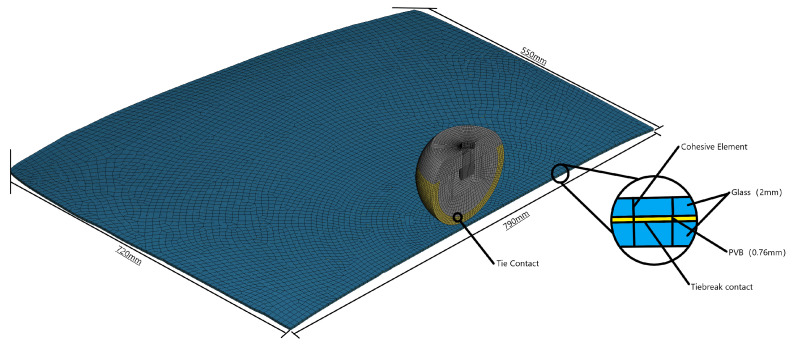
Finite element model of automobile front windshield glass (half-model).

The specific car front windshield used as the test specimen is the ELANTRA (Beijing Hyundai Elantra). In order to simplify the calculation, a semi-model is adopted for modeling, and the finite element model established is shown in Figure 17. This windshield consists of upper and lower glass layers with a thickness of 2 mm each, while the middle layer is made of a PVB film with a thickness of 0.76 mm. Furthermore, to accurately simulate real-life human–vehicle collision accidents, the automobile windshield test specimen is bonded with glass glue and fixed to an iron frame. The dummy head’s built-in acceleration sensor is utilized to measure the acceleration curve of the pedestrian head during the impact process. Additionally, the high-speed camera is employed to record the failure process of the automobile windshield glass under the impact load of the dummy head. This combination of measurements and recordings facilitates the analysis of the dynamic failure response of the automobile windshield glass.

For the purposes of the subsequent simulation analysis, this paper selects impact tests with a fixed impact angle of $90^{\circ }$ and impact velocities of 6.6 m/s. These tests serve as the main objects of the simulation analysis evaluation.

### 5.2. Results Analysis

**Figure 18:**
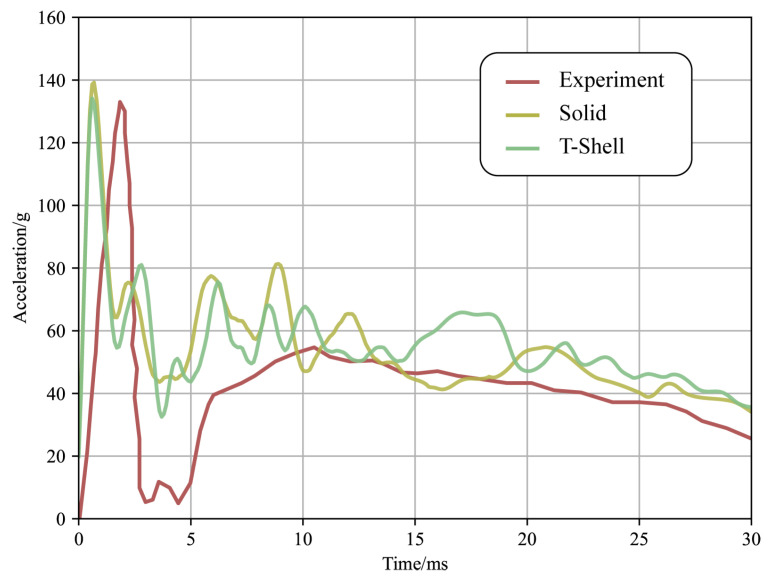
Acceleration curve of the dummy head.

**Figure 19:**
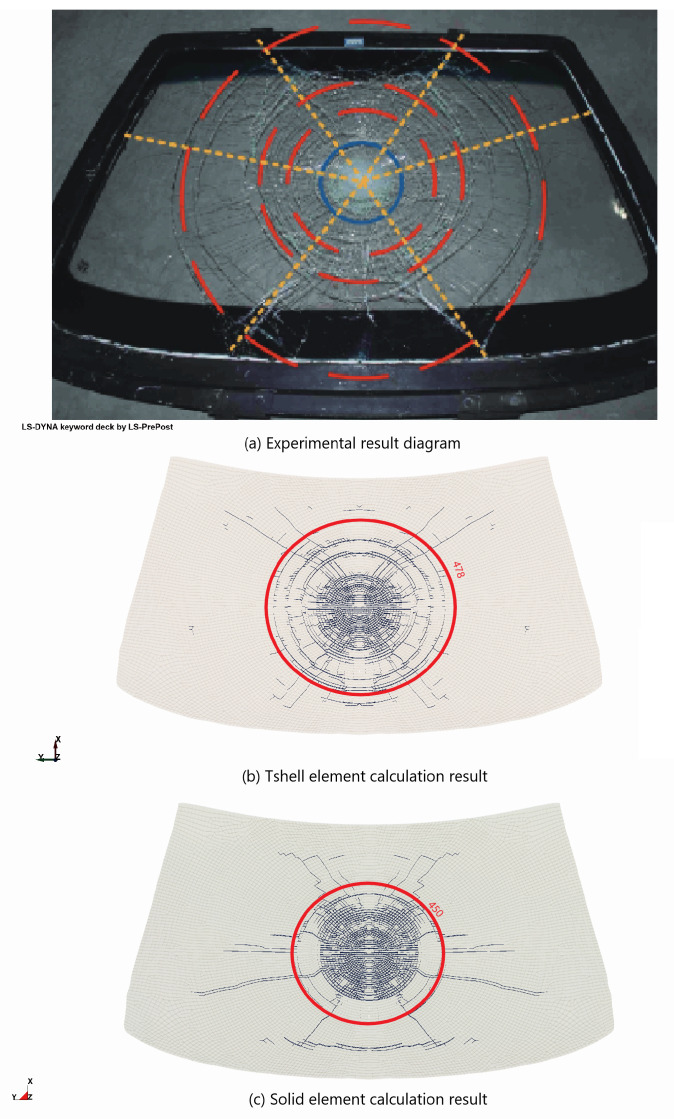
Comparison of front windshield crack effect.

First, the calibration of the acceleration curve was conducted, as shown in Figure 18, to verify the efficiency of the model. It was observed that the peak acceleration of the solid element was higher than the experimental value due to shear locking, whereas the Tshell element demonstrated better performance in this aspect. The specific effect of crack growth is depicted in Figure 19, and the experimental results and solid element calculations were obtained from [30].

**Figure 20:**
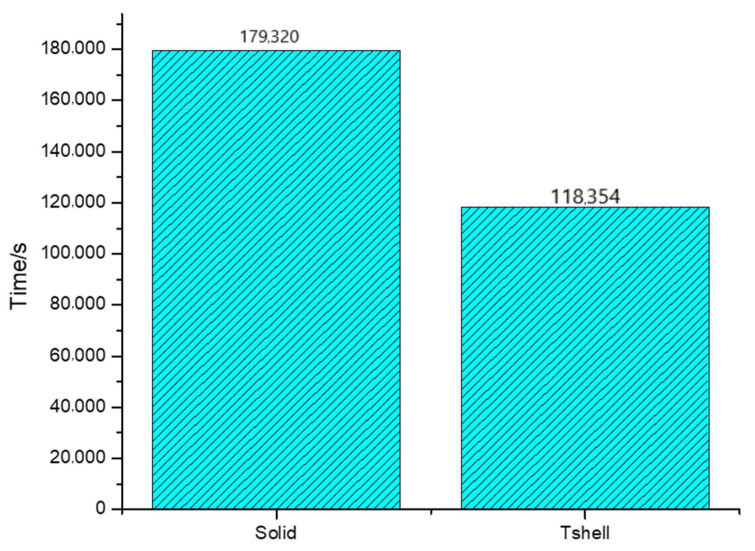
Comparison of time spent calculating the impact damage of front windshield glass.

Subsequently, Figure 20 shows a comparison of the calculation times of the Tshell and solid elements, leading to the conclusion that the Tshell element exhibits a shorter calculation time. This demonstrates that the Tshell element holds significant practical application value.

## 6. Parametric Study

### 6.1. The Effect of the Thickness Ratio of Each Layer

In an effort to streamline the problem, prior studies often made the assumption that the upper and lower layers of glass had identical thicknesses. However, for the purposes of scientific rigor and structural optimization, it is imperative to reasonably question and verify this assumption. This paper aimed to investigate the impact resistance of glass by keeping the total thickness of windshield glass constant and exploring the influence of the thickness ratio of each individual layer. Moreover, by eliminating shear locking, the feasibility of the calculation results can be significantly enhanced.

Several previous studies have endeavored to examine the thickness ratio of laminated glass layers and the energy absorption characteristics of the PVB layer. Some of these studies have even demonstrated that the inclusion of PVB layers can assist in mitigating peak acceleration. These findings indicate that the PVB layer benefits from having a thicker upper laminated glass layer, as it allows for better reflection of the ductility of its material, thereby leading to improved energy absorption properties and a reduction in the peak acceleration resulting from impacts. Interestingly, these conclusions appear to challenge common intuition.

The specific thickness parameters were set as shown in Table 2.

**Figure 21:**
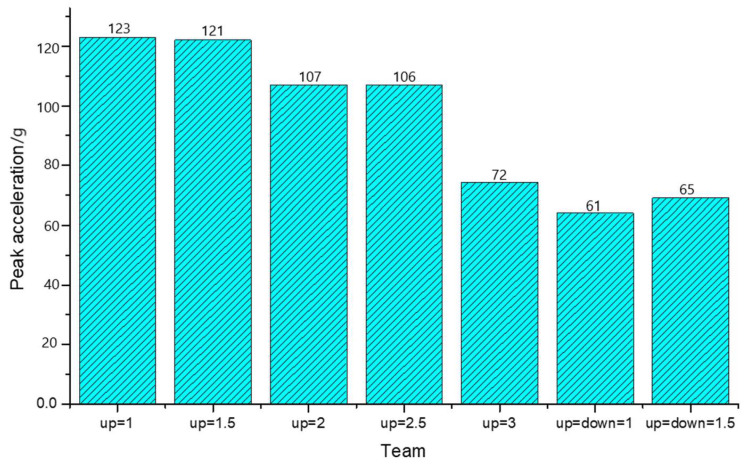
Histogram of the peak acceleration for different thicknesses of the upper glass.

It can be readily observed in Figure 21 that the peak acceleration of the glass decreased as the thickness ratio of the upper glass increased, which aligns with the findings of theoretical inference. Moreover, Figure 21 reveals an important point to consider: when the thickness ratio of the intermediate PVB layer increased, the peak acceleration showed a tendency to decrease, deviating from the conclusions of previous studies. This inconsistency can be attributed to the fact that this study focused on the condition of maintaining the same total thickness. Consequently, as the PVB layer became thicker, the overall thickness of the glass decreased, leading to a reduction in the peak acceleration.

**Figure 22:**
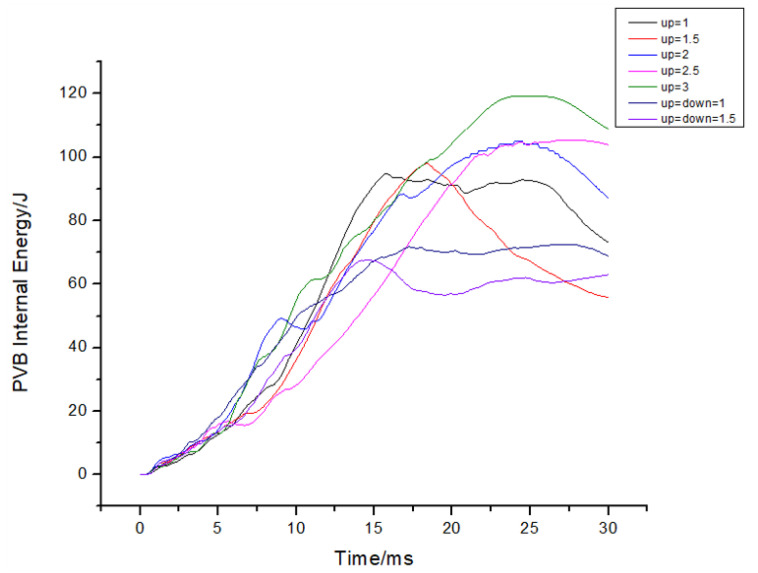
The variation of energy in the PVB layer over time under different thickness ratios.

Moreover, it can be observed that as the thickness of the upper glass increased, there was a corresponding change in the relative position of the PVB material, as depicted in Figure 22. This led to an increase in the peak energy of the PVB material and a delay in the peak time. Additionally, it is worth noting that the impact of the PVB material thickness on the peak internal energy was not linear. Instead, it exhibited a trend of initially decreasing and then increasing.

### 6.2. The Effect of the Number of Layers

**Figure 23:**
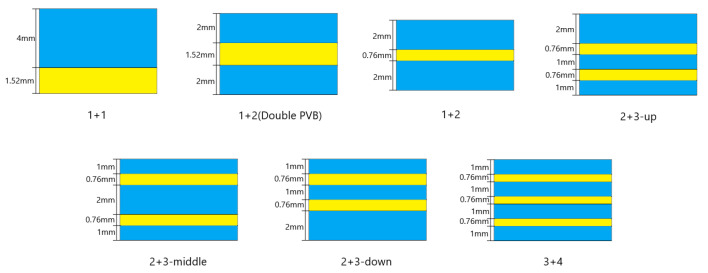
Distribution diagram of the number of layers.

Multilayer laminated glass is extensively employed in various applications, such as glass curtain walls of buildings, bulletproof car glass, and the front windshields of high-speed trains. Typically, multilayer laminated glass is associated with increased thickness. However, few studies have specifically compared the impact resistance resulting from merely increasing the thickness versus increasing the number of layers. In this study, the total thickness of the front windshield glass was kept constant, and the impact resistance of the windshield glass was investigated by varying the number of layers. This is illustrated in Figure 23.

**Figure 24:**
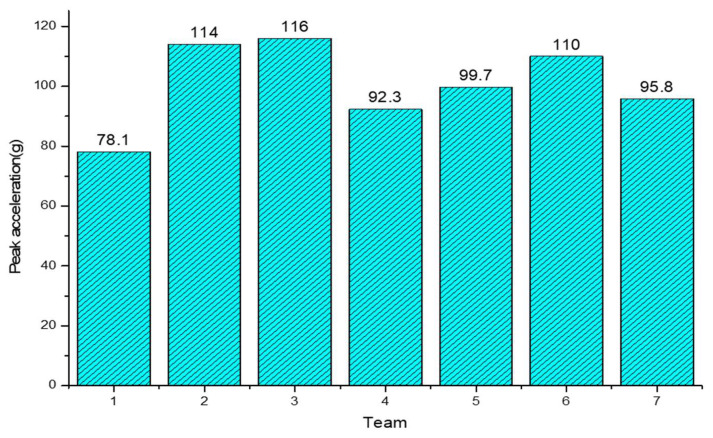
The trend of the peak acceleration with the number of layers.

The following conclusions can be drawn from the analysis of Figure 24: Firstly, an increase in PVB thickness resulted in an increase in the peak acceleration, which aligns with the findings of previous studies. Additionally, this study also revealed that increasing the number of PVB layers slightly reduced the peak acceleration. However, it is important to note that, based on the data, the influence on the peak acceleration was minimal.

Secondly, when comparing the three groups of examples involving two layers of PVB and three layers of glass, as depicted in Figure 24, it can be concluded that placing the PVB layer in the lower position effectively reduces peak acceleration. This conclusion further supports the research findings presented in the previous section.

Meanwhile, increasing the number of layers can effectively reduce the peak acceleration. However, a reasonable arrangement of two layers of PVB and three layers of glass can result in a lower peak acceleration than four layers of glass.

**Figure 25:**
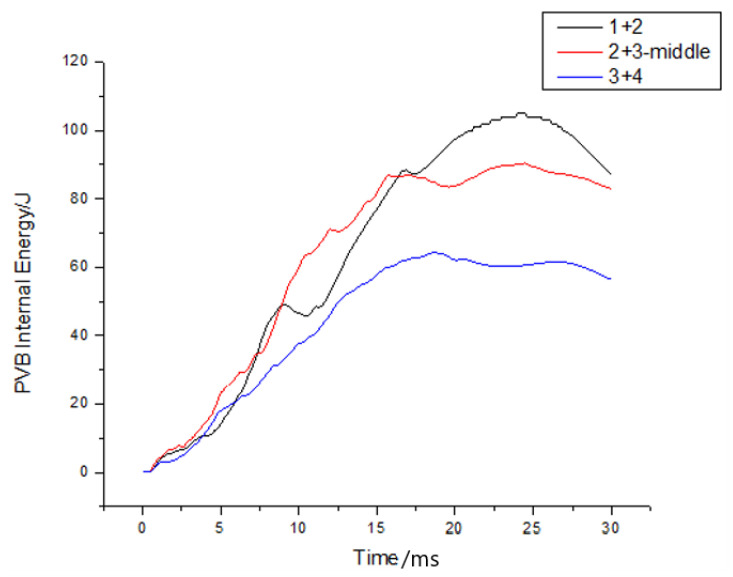
PVB internal energy histories for cases with varying numbers of layers.

**Figure 26:**
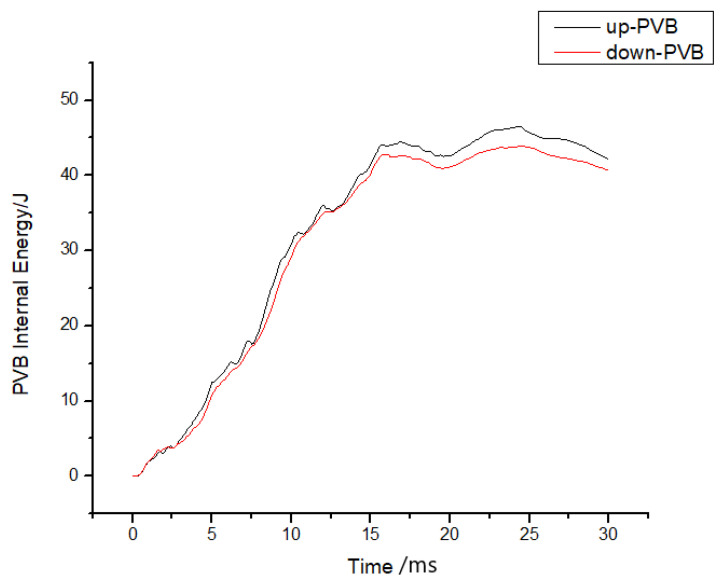
PVB internal energy histories for cases comparing the upper and lower PVB layers.

Moreover, the impact resistance of windshield glass has been widely studied in the context of multilayer laminated glass. Nonetheless, contrary to common belief, this investigation has uncovered that apart from double-layer glass, which exhibits a higher peak acceleration, increasing the number of layers results in a decline in the peak acceleration. This phenomenon can be attributed to the positioning of the PVB layer, as discussed earlier, which is lower in both single- and multilayer glass, thus diminishing the peak acceleration. This trend is evident in Figure 25, which depicts the energy history of each PVB layer. Furthermore, when examining a specific calculation example, such as calculation case No. 5, and analyzing the upper and lower PVB internal energy curves, it becomes apparent that the upper PVB internal energy surpassed that of the lower PVB. This indicates that the peak internal energy of the PVB layer was influenced not only by its location but also by the impact side, where the peak internal energy was higher compared to the non-impact side, as illustrated in Figure 26.

**Figure 27:**
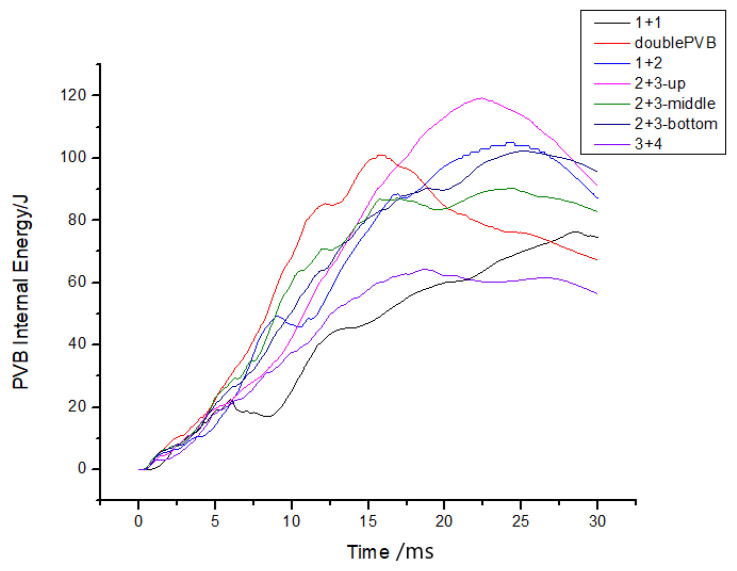
PVB internal energy histories for different examples.

The PVB layer’s internal energy was simultaneously extracted for each group of examples, as shown in Figure 25. The internal energy curves were compared, revealing that an increase in the number of layers led to a decrease in the peak PVB internal energy, with an advanced peak time. Moreover, increasing the thickness of the PVB did not result in an increase in the peak value of the PVB internal energy, as shown in Figure 27, but rather advanced the time required to reach the peak value.

### 6.3. The Effect of Curvature

In order to enhance aesthetic appeal and impact resistance, the front windshield glass of cars is commonly designed with a specific curvature or arc. The method presented in this paper offers a comprehensive analysis of the effect of this curvature, thanks to its effective resolution of the shear-locking issue. Hence, a series of illustrative examples are employed in this study, and the corresponding results are documented in Table 3.

**Figure 28:**
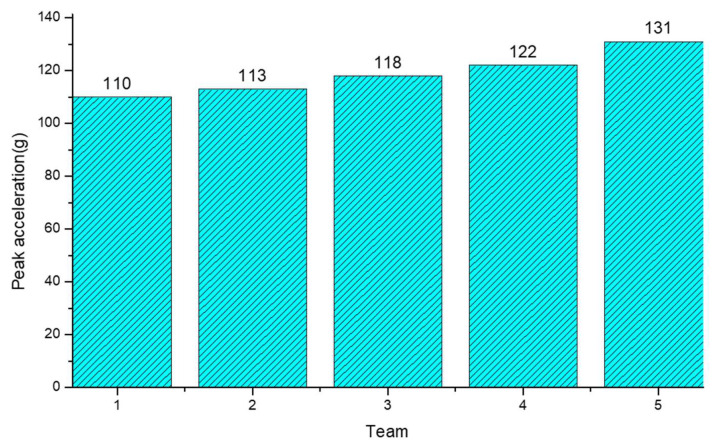
Histogram of peak acceleration under different curvatures.

Upon comparison, it can be observed (Figure 28) that the impact of the curvature on the peak acceleration was not significant. However, it is notable that as the curvature increased, the curved properties of the windshield glass became more apparent. Consequently, the windshield glass exhibited enhanced rigidity, resulting in a larger peak acceleration value for the dummy-head model.

**Figure 29:**
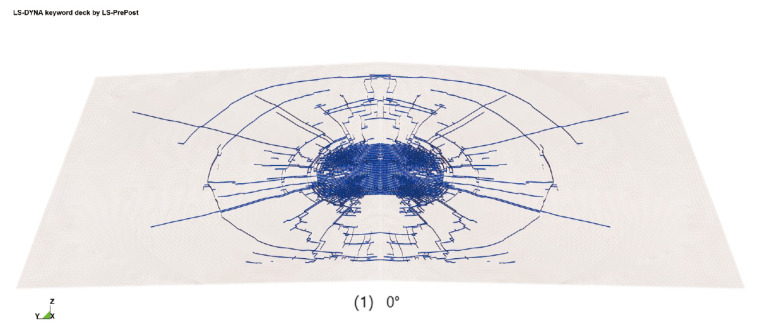

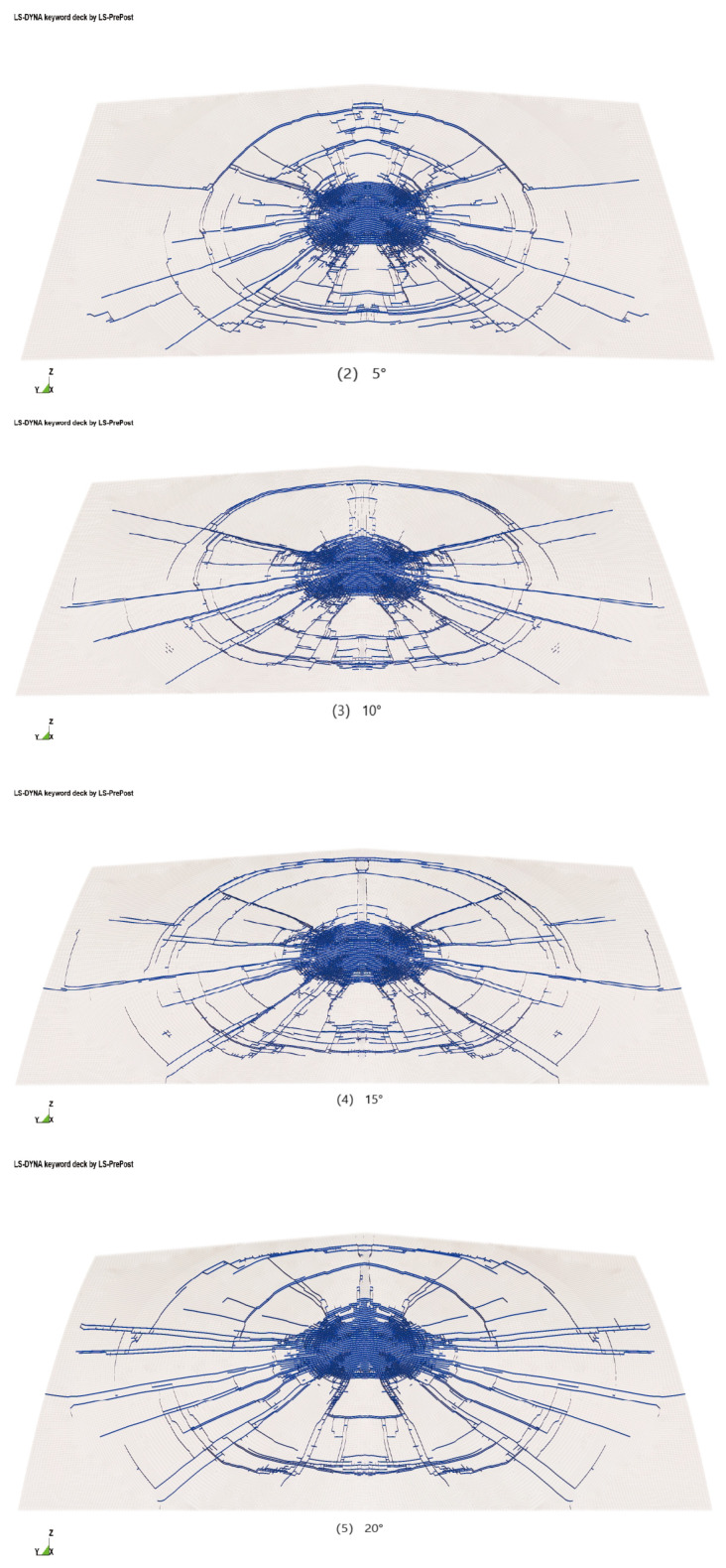
Comparison diagram of cracks under different curvatures.

However, based on the image sequence of the glass crack displayed in Figure 29, it can be inferred that increasing the curvature of the glass would indeed result in a slight elevation in the peak acceleration of the dummy-head model. Moreover, it would lead to a more extensive distribution of cracks in the glass, with the outermost ring of cracks exhibiting a larger diameter. This indicates a more thorough destruction of the glass, resulting in a more substantial absorption of energy.

## 7. Conclusions

Tshell and cohesive elements embedded in LS-DYNA were utilized to investigate the impact failure of glass. The following conclusions can be drawn:

(1) The effectiveness of combining Tshell and cohesive elements is validated using a DCB example. It is found that this combination yields good calculation results, particularly in the field of glass impact damage. These findings lay a theoretical foundation for future simulation examples and similar studies.
(2) Concerning the issue of different thickness ratios of glass layers, an interesting observation emerges: an increase in the thickness of the upper glass layer correlates with a decrease in the peak acceleration of the dummy-head model. This phenomenon can be attributed to the positioning of the PVB layer in the lower part, which allows it to withstand a greater tensile stress wave. Consequently, the ductility of the PVB material is fully utilized, resulting in a reduction in the peak acceleration of the dummy-head model.
(3) The Tshell and cohesive elements utilized in this study effectively address the issue of shear locking, thereby bolstering the dependability of our findings. When a curvature exists, the arched configuration of the windshield promotes higher resistance against impact, consequently leading to an increased peak acceleration. On the whole, the curvature insignificantly influences the peak acceleration.

In addition to the application scenarios mentioned in this article, the occurrence of bird impacts on windshields represents a significant traffic hazard. Birds frequently land on roads or protective belts and attempt to escape upon perceiving an approaching vehicle. Unfortunately, due to the velocity of the vehicle, collisions often transpire. Such incidents can give rise to a variety of severe consequences.

Primarily, the impact of a bird striking the windshield has the potential to splinter the glass, thus posing an inherent risk of injury to both drivers and passengers. Additionally, the resulting fractures in the glass can obstruct the driver’s line of sight, while the presence of scratched glass fragments can impair the driver’s ability to navigate accurately. Consequently, situations may arise where vehicles roll over or collide with guardrails, thereby causing secondary injuries. Moreover, the presence of glass cracks can disrupt the normal function of vehicles, thereby impeding travel plans. The ramifications of this impairment are intensified in scenarios entailing racing or emergency situations.

## Figures and Tables

**Table 1:** Material models and parameters.

Name	Impactor	Glass	PVB and Rubber
Material model	Rigid	Elastic	Mooney–Rivlin Rubber
Density/(kg/m${}^{3}$)	——	2500	1100
Elastic/GPa	207	74	——
Poisson’s ratio	0.27	0.2	0.49

**Table 2:** Table of layer thicknesses of each group study (unit:mm).

	1	2	3	4	5	6	7
upper	1	1.5	2	2.5	3	1	1.5
PVB	0.76	0.76	0.76	0.76	0.76	2.76	1.76
lower	3	2.5	2	1.5	1	1	1.5

**Table 3:** Example settings for the parametric study of the curvature.

	1	2	3	4	5
curvature	0°	5°	10°	15°	20°

## Data Availability

The data presented in this study are available on request from the corresponding author.

## Nomenclature

β	the mixed factor of the crack form
Δu	the displacement increment in the period of $\left[t_{n},t_{n+1}\right]$
$\Delta u^{def}$	pure deformation in the global coordinate system
$\Delta u^{rot}$	pure rotation in the global coordinate system
Δ	the relative displacement of points at the crack interface in this region
$\delta^{F}$	the ultimate displacement
$\Omega_{x}^{\prime }$	virtual configuration at time x
$\Omega_{x}$	configuration at time x
$\sigma_{x}$	stress at time x
$\varepsilon_{x}$	strain at time x
*A* and *B*	material parameters of PVB

*B*	strain matrix
$C_{t}^{max},C_{n}^{max}$	the maximum cohesive strength for mode I and mode II
*E*	Young’s modulus
$E^{\prime }$	normal or inplane stiffness
$f^{in}$	Internal force of element
$G_{C}$	the energy release rate of type I or type II cracks
$G_{I}$	the critical energy release rate for mode I
$I_{1}$, $I_{2}$, and $I_{3}$	invariants related to R
*J*	Jacobian matrix
*k*	stiffness
$Q_{MAX}$	the tensile strength T of type I crack or shear strength S of type II crack
$R_{n+1/2}$	transformational matrix
Ri	the orthogonal transformation matrix
*T*	cohesive force
*u*	the ultimate displacement in the normal or tangential direction
*V*	element field
*v*	Poisson’s ratio
XMU	the mixed-mode failure criterion index, where XMU is less than zero
$x_{n}$ and $x_{n+1}$	the spatial coordinates of the two configurations in global Cartesian coordinates,
	which are represented using local coordinates
R	the right Cauchy–Green strain tensor
